# Efficacy and safety of semaglutide in glycemic control, body weight management, lipid profiles and other biomarkers among obese type 2 diabetes patients initiated or switched to semaglutide from other GLP-1 receptor agonists

**DOI:** 10.1007/s40200-021-00899-9

**Published:** 2021-09-25

**Authors:** Aki Okamoto, Hirohide Yokokawa, Tomoko Nagamine, Hiroshi Fukuda, Teruhiko Hisaoka, Toshio Naito

**Affiliations:** 1 OKM Okamoto Internal Medicine Clinic, Tokyo, Japan; 2 Department of General Medicine, Faculty of Medicine, Juntendo University, 2-1-1 Hongo, Bunkyo-ku, Tokyo, 113-8421 Japan; 3 Division of Diabetes, Endocrinology and Metabolism, Department of Medicine, Nippon Medical School, Tokyo, Japan

**Keywords:** Type 2 diabetes, Obesity, Incretin, Glucagon-like peptide-1 receptor agonists, Body weight

## Abstract

**Purpose:**

Evidence of the efficacy and safety of semaglutide among patients with type 2 diabetes who were initiated on or were switched to semaglutide from other GLP-1 RAs remains limited. The objective of this study was to investigate the short-term effects of switching to semaglutide from other GLP-1 RAs.

**Methods:**

This retrospective cohort study evaluated patients with type 2 diabetes who were initiated on or were switched to semaglutide due to poor diabetes control with other GLP-1 RAs or other medications, or obesity. HbA1c, body weight, serum creatinine, serum uric acid, parameters of lipid metabolism, and parameters of liver function were measured before and 6 months after administration of semaglutide.

**Results:**

A total of 50 patients were registered in the study. After switching to semaglutide (n = 43), HbA1c and body weight significantly decreased (*p* < 0.01, *p* < 0.01), respectively. The same findings were observed in semaglutide-naïve patients (*p* = 0.04, *p* < 0.02) (n = 7). Serum uric acid, total cholesterol, triglycerides, and urinary albumin-creatinine ratio decreased significantly as well (*p* = 0.04, *p* = 0.04, *p* = 0.02, p = 0.04), whereas serum creatinine did not change significantly (*p* = 0.51).

**Conclusions:**

Semaglutide showed excellent efficacy, even in patients switched from other GLP-1 RAs. Semaglutide appears to be a promising agent for blood glucose and body weight control in obese type 2 diabetes mellitus patients and could be more potent in treating type 2 diabetes than existing GLP-1 RAs.

## Introduction

Type 2 diabetes is a chronic disease caused by impaired insulin secretion due to β-cell dysfunction and insulin resistance in peripheral tissues. Homeostatic hyperglycemia causes microangiopathy (neuropathy, retinopathy, and nephropathy) and carries a risk of macroangiopathy including cardiovascular disorders [1, 2]. Complications of these vascular disorders are associated with a decrease in patient quality of life, a decrease in healthy life expectancy, and economic disadvantages [3]. Furthermore, because obesity is a risk factor for macroangiopathy and associated with the pathogenesis of type 2 diabetes, obese type 2 diabetes patients require more intensive glycemic control and body weight loss [4]. Lifestyle changes (diet and exercise) along with medication can be effective on treatment [5, 6]. However effective medication is essential in severely diabetic patients.

Incretin, which consists of glucagon-like peptide-1 (GLP-1) and glucose-dependent insulinotropic polypeptide (GIP), is a small intestine hormone that stimulates the secretion of insulin upon feeding [7]. In type 2 diabetes, excessive secretion of glucagon also plays a role in the mechanism of hyperglycemia [8]. Because incretin inhibits glucagon secretion [7] in addition to promoting insulin secretion, its application as a drug for type 2 diabetes has been aggressively pursued. However, as the reactivity of GIP in β-cells is decreased in type 2 diabetes [9], efforts to develop GLP-1–related drugs have led to the use of GLP-1 receptor agonists (GLP-1 RAs) in the treatment of type 2 diabetes.

GLP-1 RAs have a variety of effects in addition to hypoglycemic effects because they achieve concentrations higher than those under physiologic conditions. The most characteristic effect of GLP-1 RAs is weight loss, which is thought to be related to delayed gastric emptying and central anorexigenic effects [10]. Delayed gastric emptying is considered more important than insulin secretion in the control of postprandial hyperglycemia [11]. Semaglutide is a newly introduced once-weekly GLP-1 RA formulation that became clinically available in Japan in June 2020. Semaglutide showed excellent hypoglycemic and weight-reducing effects in the SUSTAIN studies series [12, 13]. Unlike conventional GLP-1 RAs, semaglutide directly translocates to the brain stem, lateral septal nucleus, and hypothalamus, where it exerts central neurologic effects [14].

Available data suggest that semaglutide is an effective therapeutic option for obese type 2 diabetes patients exhibiting insufficient weight loss in treatment with conventional GLP-1 RAs. However, evidence regarding the efficacy and safety of semaglutide in patients with type 2 diabetes in Japan who were initiated on or switched to semaglutide from other GLP-1 RAs remains limited. The primary objective of this study, therefore, was to investigate the short-term effects of switching to semaglutide from other GLP-1 RAs in order to explore the therapeutic positioning of semaglutide as a treatment option for type 2 diabetes in actual clinical settings.

## Methods

### Study design and participants

This study was a retrospective, single-center, cohort study conducted between July 2020 and February 2021. The study participants were drawn from among type 2 diabetes patients attending the Okamoto Internal Medicine Clinic, Tokyo, Japan. Diabetic patients who were switched to semaglutide from other GLP-1 RAs due to poor glycemic control (HbA1c ≥ 6.5%) and/or obesity (body mass index [BMI] ≥ 25 kg/m^2^) were registered in the study. The study did not have a control group and this was a one arm study. The dose of semaglutide was started at 0.25 mg once weekly and increased to 0.5 mg once weekly after 4 weeks. If the efficacy of 0.5 mg once weekly for ≥ 4 weeks was insufficient, the dose was increased to 1.0 mg once weekly. The dosage and administration of hypoglycemic agents other than GLP-1 RAs that had been administered prior to treatment with semaglutide was altered depending on the status of blood glucose control. The study inclusion criteria were as follows: 1) age ≥ 20 years during the study period, 2) treated with an existing GLP-1 RA for ≥ 3 months before receiving semaglutide or naïve to semaglutide, and 3) HbA1c ≥ 6.5% with BMI ≥ 25.0 kg/m^2^. The exclusion criteria were as follows: 1) a history of pancreatitis, 2) severe gastrointestinal disorders such as severe gastroparesis, 3) severe hypoglycemia with conventional therapy, 4) pregnancy, and 5) severe renal dysfunction.

### Parameters

The date semaglutide first prescribed was defined as the baseline. Age, sex, height, and body weight at baseline were recorded. BMI was calculated by dividing weight (kg) by height (m) squared. Blood and urine samples were collected after overnight fasting, and body weight, blood pressure, and heart rate were recorded at baseline and at 3 and 6 months after initiation of semaglutide. Blood samples were collected after overnight fasting. C-peptide was measured at only baseline by chemiluminescence immunoassay. HbA1c was measured by high-performance liquid chromatography and expressed according to National Glycohemoglobin Standardization Program values. Serum creatinine, serum uric acid, triglycerides, high-density-lipoprotein cholesterol (HDL-C), and total cholesterol (TC) were also measured by an enzyme method, uricase-POD method, enzyme method, direct method, enzyme method, enzyme method, respectively. Low-density-lipoprotein cholesterol (LDL-C) was estimated using the Friedewald equation ([TC] – [HDL-C] – [TG/5]). The urinary albumin to creatinine ratio (ACR) was also determined by a turbidimetry and enzyme method. To evaluate the safety of semaglutide, aspartate transaminase (AST), alanine aminotransferase (ALT), gamma-glutamyl transpeptidase (γ-GTP), white blood cell count, red blood cell count, hemoglobin content, hematocrit, albumin, lactate dehydrogenase (LDH) and blood urea nitrogen were also measured by a modified JSCC reference method, flow-cytometry method, May-Giemsa heavy staining method, SLS-Hb method, pulse wave method, urease GLDH method, modified IFCC reference method, respectively. The estimated glomerular filtration rate (eGFR) was calculated using the following equations:

$$\begin{array}{l}\mathrm{Males}:\;\mathrm{eGFR}=194\times\mathrm{serum}\;\mathrm{creatinine}^{-1.094}\times\;\mathrm{age}^{-0.287}\\\mathrm{Females}:\;\mathrm{Male}\;\mathrm{value}\times0.739\end{array}$$

### Endpoints

The primary endpoints were changes in HbA1c level and body weight from baseline to 3 and 6 months. The main secondary endpoint was achievement of HbA1c < 6.5% at 6 months after initiation of semaglutide. In addition, changes in the dosage and administration of concomitant hypoglycemic agents were also assessed as secondary endpoints. As an exploratory aim, changes in lipid metabolism parameters and ACR were analyzed. Adverse events, including changes in laboratory values, for which a relationship to semaglutide could not be ruled out were defined as adverse drug reactions.

### Statistical analysis

Parametric variables are expressed as mean ± standard deviation in the text, tables and figure. Non-parametric variables are expressed as the median (25th percentile, 75th percentile). Data exhibiting a non-normal distribution are expressed as median and interquartile range. Comparisons between baseline and 3 and/or 6 months were estimated using paired *t*-tests or Wilcoxon rank-sum tests. Pearson’s correlation coefficients were calculated for changes in HbA1c and other parameters, including body weight. EZR software, ver. 1.4., and Mediating 1 R, ver. 3.5.2 (The R Foundation for Statistical Computing, Vienna, Austria) [15], were used for analyses, and a two-sided *p*-value of < 0.05 was considered statistically significant.

The study plan was approved by the Okamoto Internal Medicine Clinic Committee and complied with the Helsinki Declaration and the “Ethical Guidelines for Medical Research Involving Human Subjects”. The research protocol was reviewed and approved by the Ethics Committee of Juntendo University (no. 2020311), and written informed consent was obtained from all participants.

## Results

During the study period, 43 patients were switched from a conventional GLP-1 RA to semaglutide, and 7 patients were naïve to semaglutide; all patients agreed to be registered in the study. All 50 patients continued with semaglutide for at least the 6-month observation period. The following GLP-1 RAs were administered before semaglutide in 43 patients (switching to semaglutide group); dulaglutide in 24 patients (mean dose: 0.75 mg), liraglutide in 18 patients (1.60 ± 0.33 mg), and exenatide in 1 patient (10 μg). All 7 patients not receiving a GLP-1 RA before administration of semaglutide (naïve to semaglutide group) were treated with sodium-glucose cotransporter-2 (SGLT2) inhibitors. The dose of semaglutide after 6 months was 0.92 ± 0.19 mg in all patients.

Table 1 lists the characteristics of each group as well as the 50 patients treated with semaglutide. In the 50 patients, males composed 42.0% of the study group, and the mean age was 51.3 ± 11.0 years. Mean BMI was 32.2 ± 6.2 kg/m^2^, and insulin secretion was relatively preserved, as evaluated by C-peptide levels.

**Table 1:** Characteristics of participants at baseline

	Total (n = 50)		Switching to semaglutide (n = 43)		Naïve to semaglutide (n = 7)	
Male	21	(39.5)	17	(42.0)	4	(57.1)
Age (years)	51.3	(11.0)	50.2	(10.8)	57.7	(10.8)
BMI (kg/m^2^)	32.2	(6.2)	31.9	(6.3)	31.9	(6.3)
Body weight (kg)	87.7	(17.9)	86.5	(18.8)	95.3	(8.0)
HbA1c (%)	6.78	(0.73)	6.72	(0.62)	7.19	(1.21)
C-Peptide (mg/dL)	2.7	[1.9, 3.9]	2.6	[1.8, 3.6]	4.3	[3.1, 6.8]
Uric acid (mg/dL)	4.90	(1.03)	4.85	(1.05)	5.21	(0.94)
Total cholesterol (mg/dL)	183.8	(41.0)	184.0	(41.6)	182.4	(40.3)
HDL cholesterol (mg/dL)	54.9	(11.9)	54.9	(11.7)	55.0	(13.9)
LDL cholesterol (mg/dL)	93.9	(26.2)	94.7	(24.9)	89.0	(35.1)
Triglyceride (mg/dL)	133.5	[92.5, 196.0]	135.0	[93.0, 183.0]	132.0	[88.5, 237.5]
Serum creatinine (mg/dL)	0.69	(0.16)	0.68	(0.16)	0.74	(0.12)
eGFR (mL/min/1.73m^2^)	84.3	(22.6)	85.5	(23.2)	76.4	(17.6)
ACR (mg/g·CRE)	14.0	[6.7, 22.8]	11.3	[6.6, 20.0]	32.4	[17.7, 44.1]

Figures are shown as number (percentage), mean (standard deviation, SD) or median [25th percentile, 75th percentile]

*BMI* body mass index, *HDL* high density lipoprotein, *LDL* low density lipoprotein, *eGFR* estimated glomerular filtration rate, *ACR* albumin creatine ratio

### Changes in HbA1c and body weight

**Fig. 1:**
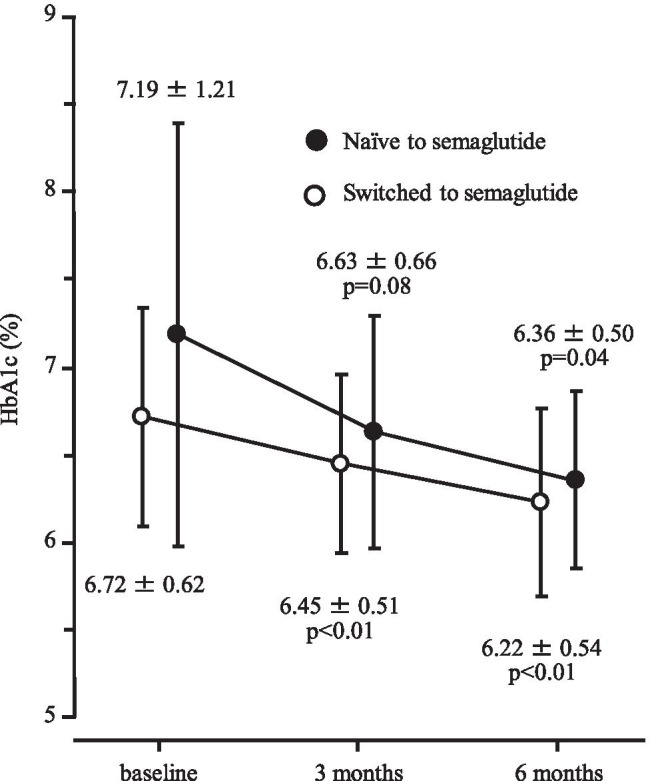
Change in HbA1c after 3 and 6 months of follow-up

**Fig. 2:**
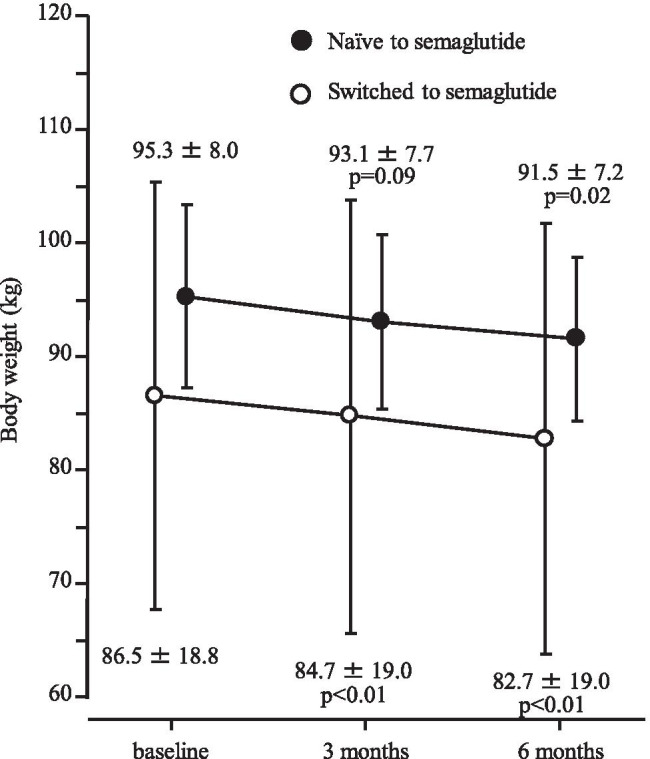
Change in body weight after 3 and 6 months of follow-up

In 43 patients in switching to semaglutide group, HbA1c level decreased significantly, from 6.72 ± 0.62% at baseline to 6.45 ± 0.51% after 3 months (p < 0.01) and 6.22 ± 0.54% after 6 months (*p* < 0.01) as shown in Fig. 1. Similarly, body weight declined significantly, from 86.5 ± 18.8 kg to 84.7 ± 19.0 kg after 3 months (p < 0.01) and 82.7 ± 19.0 kg after 6 months (p < 0.01) (Fig. 2). The proportion of patients who achieved HbA1c < 6.5% at 6 months was 60.6%.

In the 7 semaglutide-naïve patients, HbA1c level decreased significantly, from 7.19 ± 1.21% at baseline to 6.36 ± 0.50% after 6 months (*p* = 0.04, Fig. 1). Body weight also declined significantly, from 95.3 ± 8.0 kg to 91.5 ± 7.2 kg (*p* = 0.02, Fig. 2). All 7 of these patients achieved HbA1c < 6.5% at 6 months.

**Fig. 3:**
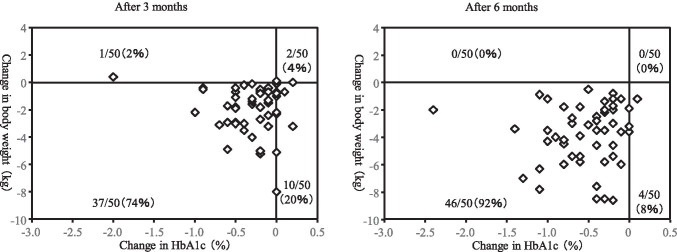
Distribution of changes in body weight and HbA1c after 3 and 6 months

Figure 3 shows the distribution of changes in body weight and HbA1c after 3 and 6 months. Decrease in both HbA1c and body weight were observed in 74% of patients after 3 months and 92% of patients after 6 months.

### Changes in the dosage and administration of concomitant hypoglycemic agents

In 19 patients receiving insulin, the insulin dose was reduced by 7.9 ± 3.7 units by study end. In 6 patients receiving sulfonylureas or glinides, dose reduction (4 patients) or interruption (2 patients) was possible.

### Factors associated with change in HbA1c

HbA1c level at baseline was significantly correlated with the change in HbA1c at 6 months after initiation of semaglutide (*r* = 0.696, *p* < 0.01). In addition, total cholesterol and triglyceride levels at baseline were significantly correlated with the change in HbA1c at 6 months (*r* = 0.312, *p* = 0.027 and *r* = 0.318, *p* = 0.03, respectively). There was no significant correlation between HbA1c change and body weight change at 6 months after initiation of semaglutide (*r* = 0.07).

### Changes in metabolic parameters

Changes in uric acid, triglycerides, total cholesterol, HDL-C, and LDL-C are summarized in Table 2. Six months after switching to semaglutide, serum uric acid, total cholesterol, and triglyceride levels were significantly decreased. In the 50 patients, ACR was also significantly lower, declining from 14.0 (6.7, 22.8) mg/g·CRE at baseline to 10.2 (6.0, 23.1) mg/g·CRE after 6 months (*p* = 0.04).

**Table 2:** Comparisons between baseline and 6 months in parameters of lipid metabolism and renal function

	Total (n = 50)			Switching to semaglutide (n = 43)			Naïve to semaglutide (n = 7)		
	Baseline	6 months	p value	Baseline	6 months	p value	Baseline	6 months	p value
Uric acid (mg/dL)	4.90 (1.03)	4.59 (1.06)	< 0.01	4,85 (1.05)	4.59 (1.11)	0.03	5.21 (0.94)	4.60 (0.80)	0.06
Total cholesterol (mg/dL)	183.8 (41.0)	168.2 (37.2)	< 0.01	184.0 (41.6)	169.9 (37.6)	0.01	182.4 (40.3)	157.7 (35.7)	0.01
HDL cholesterol (mg/dL)	54.9 (11.9)	54.0 (11.2)	0.28	54.9 (11.7)	54.4 (11.5)	0.55	55.0 (10.0)	51.7 (10.0)	0.19
LDL cholesterol (mg/dL)	93.9 (26.2)	88.4 (25.7)	0.07	94.7 (24.9)	89.5 (24.8)	0.13	89.0 (35.1)	82.0 (32.4)	< 001
Triglyceride (mg/dL)	133.5 [92.5, 196.0]	101.0 [84.0, 147.0]	< 0.01	135.0 [93.0, 183.0]	103.0 [87.5, 143.0]	< 0.01	132.0 [88.5, 237.5]	89.0 [74.5, 181.5]	0.15
Serum creatinine (mg/dL)	0.69 (0.16)	0.69 (0.16)	0.51	0.68 (0.16)	0.68 (0.15)	0.55	0.74 (01.2)	0.74 (0.15)	0.81
eGFR (mL/min/1.73m^2^)	84.3 (22.6)	84.5 (21.1)	0.84	85.5 (23.2)	85.8 (22.2)	0.86	76.4 (17.6)	76.8 (11.1)	0.91
ACR (mg/g·CRE)	14.0 [6.7, 22.8]	10.2 [6.0, 23.1]	0.04	11.3 [6.6, 20.0]	8.3 [5.8, 22.1]	0.15	32.4 [17.7, 44.1]	21.0 [18.0, 26.6]	0.16

Figures are shown as mean (standard deviation, SD) or median [25th percentile, 75th percentile]. Comparisons between baseline and 6 months were made using paired t-tests or Wilcoxon rank-sum tests

*HDL* high density lipoprotein, *LDL* low density lipoprotein, *eGFR* estimated glomerular filtration rate, *ACR *albumin creatine ratio, *6 months* 6 months after the treatment with Semaglutide

### Safety

Results of blood tests to evaluate adverse effects are summarized in Table 3. At 6 months after initiation of semaglutide, liver-related parameters (AST, ALT, γ-GTP, LDH) were significantly improved. There were no adverse events related to semaglutide except mild nausea or vomiting. No hypoglycemic events were reported during the observation period.

**Table 3:** Comparisons between baseline and 6 months in safety parameters

	Total (n = 50)			Switching to semaglutide (n = 43)			Naïve to semaglutide (n = 7)		
	Baseline	6 months	p value	Baseline	6 months	p value	Baseline	6 months	p value
Albumin (g/dL)	4.40 (0.35)	4.47 (0.32)	0.11	4.40 (0.35)	4.47 (0.32)	0,17	4.37 (0.35)	4.46 (0.35)	0.36
AST (IU/L)	32.1 (24.5)	24.5 (13.8)	< 0.01	31.0 (24.5)	22.6 (7.7)	0.01	38.9 (25.3)	36.1 (30.9)	0.72
ALT (IU/L)	42.2 (40.6)	30.5 (20.3)	< 0.01	39.4 (38.2)	27.7 (17.0)	0.01	59.4 (53.4)	47.9 (30.8)	0.50
γ-GTP (IU/L)	31.0 (20.8)	24.2 (14.2)	< 0.01	28.6 (19.4)	22.2 (12.7)	< 0.01	45.7 (24.4)	36.4 (17.5)	0.26
LDH (U/L)	191.8 (41.7)	177.9 (32.7)	< 0.01	190.1 (42.3)	175.8 (31.8)	< 0.01	202.3 (39.0)	190.9 (37.7)	0.26
White blood cell (/μL)	7088.0 (1495.9)	7002.0 (1669.1)	0.58	7067.4 (1476.4)	6986.0 (1649.0)	0.64	7214.3 (1730.5)	7100.0 (1924.4)	0.69
Red blood cell (10^4^/μL)	492.3 (48.2)	493.6 (49.9)	0.74	490.2 (50.5)	491.8 (52.5)	0.70	505.6 (30.2)	504.6 (28.4)	0.92
Hemoglobin (g/dL)	14.6 (1.8)	14.8 (1.6)	0.11	14.3 (1.8)	14.7 (1.6)	0.12	15.6 (1.3)	15.6 (1.3)	0.70
Hematocrit (%)	45.1 (4.8)	45.7 (4.5)	0.11	44.7 (5.0)	45.4 (4.7)	0.14	47.2 (2.6)	47.7 (2.7)	0.55
BUN (mg/dL)	15.6 (3.3)	15.3 (4.0)	0.56	15.5 (3.4)	15.2 (3.4)	0.57	15.8 (2.9)	15.7 (4.3)	0.91

Figures are shown as mean (standard deviation, SD). Comparisons between baseline and 6 months were made using paired t-tests

*AST* aspartate transaminase, *ALT* alanine aminotransferase, *γ-GTP *γ-glutamyl trans peptidase, *LDH* lactate dehydrogenase, *BUN* blood urea nitrogen, *6 months* 6 months after the treatment with Semaglutide

## Discussion

The results of the present study showed that switching to semaglutide enabled stricter glycemic control and promoted significant weight loss among obese patients with type 2 diabetes treated with a conventional GLP-1 RA. Also, the improvement in HbA1c with semaglutide was greater in patients with poor glycemic control (higher HbA1c at baseline), regardless of the type of GLP-1 RA used and body weight prior to initiation of semaglutide. The changes in HbA1c and body weight after switching to semaglutide were similar to those in semaglutide-naïve patients. In addition, administration of semaglutide significantly decreased uric acid levels and improved several parameters of lipid metabolism and liver function.

The Guidelines for the Treatment of Diabetes in Japan recommend increasing the dose of administered drugs or combining drugs with different mechanisms of action for targeted glycemic control [4]. However, it is necessary to avoid hypoglycemia when attempting stricter blood glucose control, especially while increasing the dose or administering additional doses [16]. In addition, elderly patients with diabetes must be treated with greater care, because these patients are more likely to be affected by hypoglycemia due to poor patient adherence [17]. In view of these considerations, switching between drugs in the same class may be worthwhile for better glycemic control and avoiding adverse effects, including hypoglycemia, and semaglutide has received considerable attention in this regard in recent years.

A retrospective analysis of clinical practice data indicated that in type 2 diabetes patients receiving the recommended dose of liraglutide or dulaglutide, HbA1c decreased by 0.65% and body weight decreased by 1.69 kg 6 months after switching to semaglutide [18]. Although the decrease in HbA1c after switching to semaglutide was greater than that in the present study, the high HbA1c of 7.9% before switching to semaglutide is considered a factor in that difference. In the present study, the baseline HbA1c of ≥ 6.5% decreased by 0.59%. In the SUSTAIN study series, semaglutide was compared with several GLP-1 RAs. Semaglutide provided more significant improvement in HbA1c than exenatide (once-weekly), dulaglutide, or liraglutide [17–19]. In addition, HbA1c decreased by 0.83% in patients receiving semaglutide for the first time. Taking these data into account, switching to semaglutide is considered a reasonable and relatively safe option for patients under GLP-1 RA treatment. In the present study, the higher the baseline HbA1c level, the greater was the decrease in HbA1c following semaglutide initiation, and no episodes of hypoglycemia were observed.

In the SUSTAIN study, semaglutide was also more cooperative than other GLP-1 RAs in terms of body weight loss assessed concurrently [19–21]. The anorexigenic mechanism of semaglutide differs from that of other GLP-1 RAs [14] and may be related to its potent weight loss effect. Weight loss associated with semaglutide is considered independent of gastrointestinal adverse reactions such as nausea and vomiting, and gastrointestinal adverse reactions are not directly related to weight loss [22]. In addition to GLP-1 RAs, SGLT2 inhibitors promote a reduction in body weight [23]. In the present study, GLP-1 RAs or SGLT2 inhibitors were administered to all patients; however, switching to semaglutide produced further body weight loss in patients who still required improvement of obesity. This finding suggests that semaglutide may have a stronger body weight–reducing effect than other GLP-1 RAs.

In the present study, lipid metabolism was improved after switching to semaglutide. Improved lipid metabolism with GLP-1 RAs was characterized by reductions in LDL-C, total cholesterol, and triglycerides in a meta-analysis [24]. However, GLP-1 RAs do not necessarily increase HDL-C levels. A combination of changes in lipid metabolism induced by GLP-1 RAs and hypoglycemia resulting from increased insulin secretion may be effective in suppressing cardiovascular events [25]. Liver function parameters also showed significant improvements, and considering the decrease in triglycerides, semaglutide was considered to have a beneficial effect on fatty liver. A meta-analysis of the therapeutic effects of GLP-1 RAs, including semaglutide, on nonalcoholic fatty liver disease and nonalcoholic steatohepatitis indicated that GLP-1 RAs reduce hepatic fibrosis on imaging and decrease ALT and γ-GTP, suggesting the possibility of treating fatty liver with GLP-1 RAs [26].

The most frequent adverse events associated with GLP-1 RAs are gastrointestinal reactions such as nausea, vomiting, and diarrhea. As these adverse events can lead to dehydration, GLP-1 RAs should be used with caution in elderly patients or those with renal impairment. Although differences in safety between GLP-1 RAs are not clear, gastrointestinal adverse reactions should be carefully considered when semaglutide is used [27]. In the SUSTAIN-6 study, which evaluated the cardiovascular safety of semaglutide, the incidence of retinopathy complications was high [28]. Rapid glycemic improvement with semaglutide may be due in part to the association with worsening diabetic retinopathy [29]; however, this should also be noted in the clinical use of semaglutide.

## Limitations

This study has several limitations worth noting. First, there may have been selection bias given the small sample size and the fact that patients were from one medical institution specialized in diabetes treatment. In addition, the study lacked a control group, and participants were receiving a heterogeneous group of concomitant glucose-lowering drugs. Therefore, application to actual clinical settings could be limited. A large-scale, multicenter controlled study will be needed to better compare our data to those from other medical settings. Second, important factors such as health behavior were not evaluated. Such factors should also be evaluated in future studies. Finally, the follow-up period of 6 months was relatively short. As a next step, cohort studies with longer follow-up periods should be conducted to assess long-term outcomes, including glycemic control.

## Conclusion

In the single-center, prospective, single-arm study, switching from other GLP-1 RAs to semaglutide resulted in a significant decrease in HbA1c and body weight. Although additional evaluation is considered necessary, semaglutide appears to be a promising agent for blood glucose and body weight control in obese type 2 diabetes mellitus patients and could be more potent than existing GLP-1 RAs for the treatment of type 2 diabetes.

## Data Availability

Data sharing is available upon the author’s request.
